# Responses of forest carbon and water coupling to thinning treatments from leaf to stand scales in a young montane pine forest

**DOI:** 10.1186/s13021-020-00159-y

**Published:** 2020-11-03

**Authors:** Yi Wang, Antonio D. del Campo, Xiaohua Wei, Rita Winkler, Wanyi Liu, Qiang Li

**Affiliations:** 1 Department of Earth, Environmental and Geographic Sciences, University of British Columbia, Okanagan, 1177 Research Road, Kelowna, BC V1V 1V7 Canada; 2 Department of Geography and Environmental Management, University of Waterloo, 200 University Ave W, Waterloo, ON N2L 3G1 Canada; 3 Research Group in Forest Science and Technology (Re-ForeST), Universitat Politècnica de València, Camí de Vera s/n, E-46022 València, Spain; 4 The British Columbia Ministry of Forests Lands, Natural Resource Operations and Rural Development, 515 Columbia St, Kamloops, BC V2C 2T7 Canada

**Keywords:** Water-use efficiency (WUE), Thinning treatments, Spatial scales, *Pinus contorta*, Gas exchange, Drought

## Abstract

**Background:**

Water-use efficiency (WUE) represents the coupling of forest carbon and water. Little is known about the responses of WUE to thinning at multiple spatial scales. The objective of this research was to use field measurements to understand short-term effects of two thinning treatments (T1: 4500 stems ha^−1^; and T2: 1100 stems ha^−1^) and the control (NT: 27,000 stems ha^−1^) on WUE at the three spatial scales (leaf level: the ratio of leaf photosynthesis to leaf transpiration; tree-level: tree growth to tree transpiration; and stand level: net primary production (NPP) to stand transpiration) and intrinsic WUEi (the ratio of leaf photosynthesis to stomatal conductance at leaf-level; and NPP to canopy conductance at stand-level) in a 16-year old natural lodgepole pine forest. Leaf-level measurements were conducted in 2017, while tree- and stand-level measurements were conducted in both 2016 (the normal precipitation year) and 2017 (the drought year).

**Results:**

The thinning treatments did not significantly affect the tree- and stand-level WUE in the normal year of 2016. However, the thinning significantly affected WUE in the drought year of 2017: T2 exhibited significantly higher tree-level WUE (0.49 mm^2^ kg^−1^) than NT (0.08 mm^2^ kg^−1^), and compared to NT, the stand-level WUE values in the thinned stands (T1 and T2) were significantly higher, with means of 0.31, 0.56 and 0.70 kg m^−3^, respectively. However, the leaf-level and stand-level WUEi in the thinned stands in the drought year were significantly lower than those in the unthinned stands. No significant differences in the leaf-level WUE were found among the treatments in 2017. In addition, the thinning did not significantly change the WUE-VPD relationships at any studied spatial scale.

**Conclusions:**

The thinning treatments did not cause significant changes in all studied WUE metrics in a normal year. However, their effects were significantly promoted under the drought conditions probably due to the decrease in soil water availability, demonstrating that thinning can improve WUE and consequently support forests to cope with the drought effects. The inconsistent results on the effects of the thinning on forest carbon and water coupling at the spatial scales and the lack of the consistent WUE metrics constraint across-scale comparison and transferring of WUE.

## Background

Water-use efficiency (WUE), representing the coupling between carbon assimilation and water consumption of vegetation, is an important parameter in modelling responses of terrestrial carbon and water cycles to climate and land cover changes [45, 65, 94, 107, 108]. At the leaf level, water-use efficiency (leaf-level WUE) is calculated as the ratio of net photosynthetic assimilation to leaf transpiration. As exchanges of CO_2_ and water vapor share the same diffusion pathway via stomata [84], intrinsic water-use efficiency (leaf-level WUEi, the ratio of net photosynthesis to stomatal conductance) is an alternative index for leaf-level carbon and water coupling, which excludes influences from evaporative demand on leaf transpiration [103]. At the individual tree level, WUE (tree-level WUE) is expressed as the ratio of tree growth (e.g., basal area increments, BAI) to whole tree transpiration [105]. And at the ecosystem level, WUE can be quantified as the ratio of gross primary production to evapotranspiration or the ratio of net primary production to transpiration (e.g., Petritsch et al. [85]. Understanding the responses of WUE at finner spatial levels at leaf and individual tree scales is essential to predicting carbon and water processes at larger spatial scales such as forest ecosystems.

Studies of forest WUE across multiple spatial scales are limited. Leaf-level studies generally focus on leaf-level WUEi, as detected from isotopic signatures of tree tissues. Isotopic leaf-level WUEi only accounts for intercellular and ambient CO_2_ concentrations, but can cover periods of low light, low temperature and dry conditions. Leaf-level WUE, is usually measured using the gas exchange method, and reflects optimal conditions for trees (near light saturation and optimal ranges of temperature) as these field measurements are most commonly made during the day [103]. Leaf-level WUEi can also be measured using the gas exchange method, however, these two methods often produce different results [103]. The discrepancy between WUEi determined by the two different methods has been ascribed to the differences in the time scale (i.e., long term and short term) [103]. Some researchers have found that leaf-level WUEi tends to be a homeostatic trait [20, 37], while others have found that leaf-level WUEi responds to changes in plant morphology [13] and to climatic controls [1]. A review by Cernusak et al. [17] suggested that environmental factors modified leaf-level WUEi, and internal physiology which varies with species, dampening its responses. When scaling up from leaf to ecosystem levels there are even more complications involved, including physical and physiological processes within the ecosystem, errors and uncertainties associated with different measurement approaches and differences in temporal and spatial resolution, all of which lead to a lack of correspondence of WUE between different spatial levels [52, 71, 73, 75]. Contrasting evidence indicates that cautions should be used in choosing the most representative indicators of carbon and water coupling and scaling them up from leaf to ecosystem levels.

Predicting WUE requires knowledge of the relationships between WUE and micrometeorological forcing at multiple spatial scales. A few leaf-level models including the equation developed by Wong and Dunin [104]; the Norman model [57, 82], the Cowan-Farquhar model [66], the Ball-Berry model [60] and the Farquhar model [35, 72] can predict the leaf-level WUE and WUEi reasonably well [57] either solely by their dependences on VPD or both the microclimatic variables and plant physiological parameters. Even though the equations embedded in those models for calculating leaf-level WUE and WUEi vary with models, all the equations show that the leaf-level WUE generally decreases while leaf-level WUEi increases with increasing VPD (Additional file 1: Table S1). The stand-level WUE calculated in the RESCAP (RESourceCAPture) model is also solely dependent on the VPD with an inversely proportional relationship [28] (Additional file 1: Table S1). Besides, there was a negative correlation between WUE at the tree and stand levels with VPD that has been either fitted with an exponential decay function or a reciprocal function (Additional file 1: Table S1). Therefore, the decreased WUE and probably an increased WUEi corresponding to an increasing VPD is generally expected at various spatial scales. Moreover, the relationship between WUE and VPD is also dependent on light intensity as stomata can react to decreases in photosynthesis under lower light intensity by closing, thus reducing transpiration [10, 62]. Water availability (e.g., soil water contents) can also influcence the sensitivitity of WUE to climatic variables through its effects on stomatal behavior [46]. Given the evidences that thinning changed the sensitivities of tree growth or tree transpiration to microclimates [53, 56, 68, 95], it is generally expected that forest thinning treatments would affect the WUE-microclimate relationships. This issue has not been fully studied yet. To our knowledge, only one research reported that the tree isotopic WUEi became sensitive to annual precipitation after thinning [36].

**Fig. 1:**
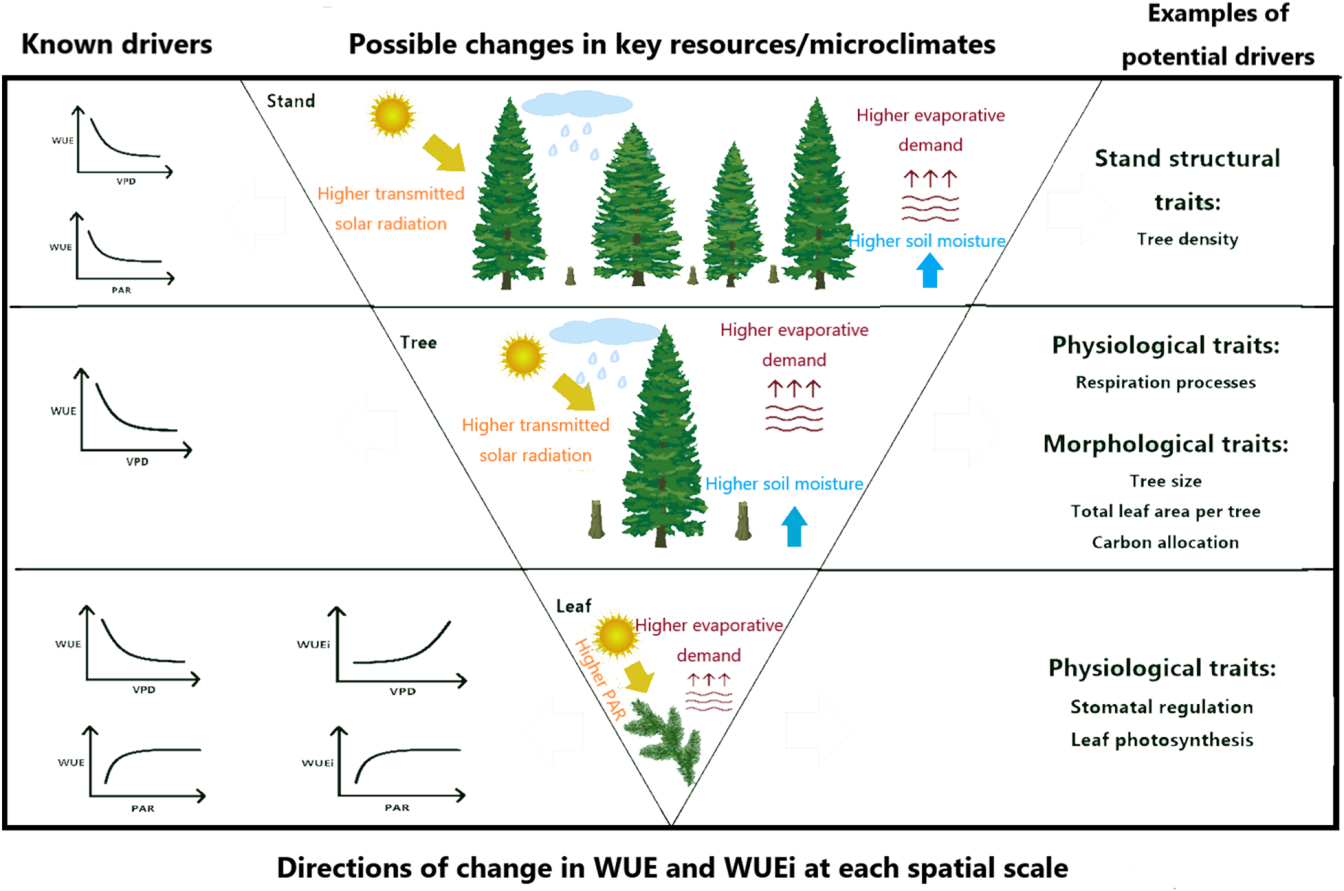
Directional hypothesis on responses of WUE to thining treatment from leaf to stand scales

To date, few studies have focused on the effects of thinning on WUE across spatial levels and under ambient conditions [62, 97]. How thinning treatments affect the responses of WUE from leaf to stand scales is largely untested. Thinning treatments usually result in a higher direct incident radiation and net rainfall reaching the ground, higher soil temperature, air temperature and wind speed, and lower air humidity within treated stands [3, 8, 19, 23, 101] (Fig. 1). At the leaf level, increased photosynthetic active radiation and atmospheric evaporative demand due to a more opened canopy created by thinning treatments can exert direct effects on leaf photosynthesis, transpiration, and stomatal conductance. But the responses of leaf-level WUE and WUEi to thinning treatments are not consistent [24, 36, 69, 81, 99]. Thinning treatments can also cause morpholocial changes in trees (e.g., promote tree size (and thus higher carbon stocks per tree) and total leaf area per tree (and thus higher tree transpiration)) and structural changes in forest stands (e.g., reduced tree density and thus lower carbon stocks per stand and a lower stand transpiration). It is unclear whether WUE and WUEi at a finer spatial level under thinning treatments show similar responses to thinning treatments at coarse spatial scales as the physiological processes operating at finer scales shift to morphological and stand structural changes operating at larger spatial scales following thinning treatments (Fig. 1).

In the separate prior study in a young lodgepole pine forest in the interior of British Columbia, Canada, we examined the effects of juvenile thinning on tree-level radial growth, sap flow velocity and stand transpiration during a drought year (2017) and a non-drought year. We found that significant differences in tree growth and sap flow velocity, between light and heavy thinning treatments, only occurred in the drought year, and the strongest response in sap flow velocity to changes in VPD occurred where trees were most heavily thinned [100]. Building on our previous work, in this study we further assess the effects of juvenile thinning on WUE across multilple spatial scales during the growing seasons of 2016 and 2017. The drought in 2017 provided an excellent opportunity of evaulating the responses of WUE to thinning treatments under these conditions. In this study, based on the theoratical relationships between WUE or WUEi (given in the “Method” section) and microclimates (as listed in Additional file 1: Table S1), we hypothesized that: (1) the juvenile thinning would decrease leaf-level WUE while increases leaf-level WUEi, if VPD would be increased by the thinning treatments, (2) there were consistent responses of WUE and WUEi to thinning treatments across three spatial scales (leaf, tree and stand) and under the drought condition; and (3) thinning would alter the sensitivity of WUE to microclimatic variables at all three spatial scales.

## Methods

### Study area and experimental design

**Fig. 2:**
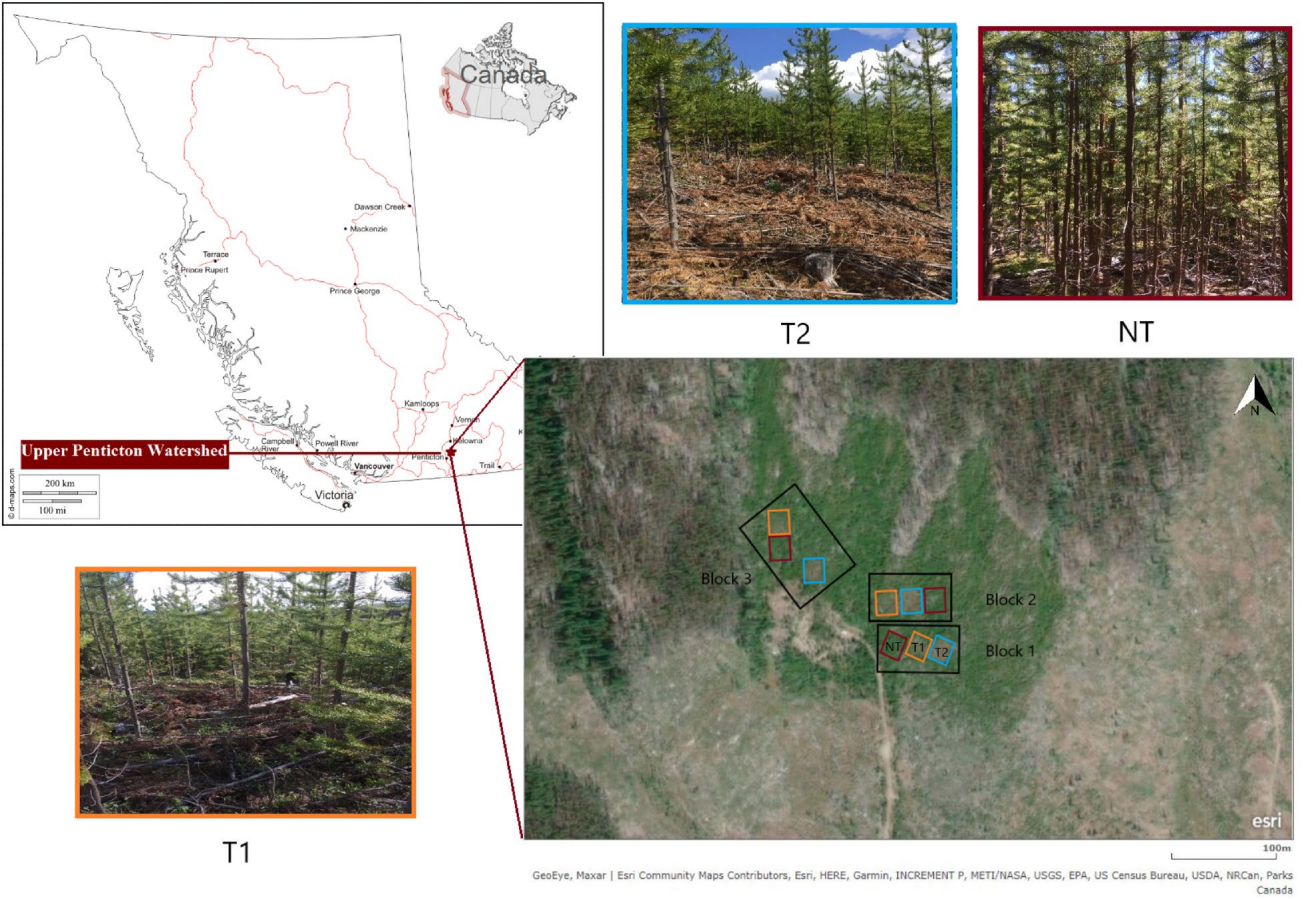
The study location, experimental layout of three blocks, and the photos of NT, T1 and T2 after the thinning treatments were applied in June 2016

The study was conducted in an even-aged 16 years old lodgepole pine *(Pinus contorta* Dougl.) forest in the Upper Penticton Watershed (UPW) in the southern interior of British Columbia, Canada (49°39′34″N, 119°24′34″W) (Fig. 2). The site is located at 1675 m a.s.l, on a south-facing slope. Soils were derived from granitic parent material, are coarse sandy-loam in texture, with low water holding capacities, and were classified as Podzols and Brunisols. Snow cover lasts from early November through to the middle of June.

In June 2016, two thinning treatments (Treatment 1 (T1: 4500 stems per ha); Treatment 2 (T2: 1100 stems per ha) and one unthinned control (NT: 27,000 stems per ha) were randomly assigned to the three plots (20 m × 20 m each) within each of the three blocks; B1, B2 and B3 (25 m × 75 m each). The slush was left on site. The understory vegetation in each plot was sparse (Fig. 2). The dominant trees DBH values range from 30 mm to 60 mm, accounting for 81.8%, 80.5% and 80.5% for NT, T1 and T2, respectively. The mean tree DBH values are 43.7, 43.5 and 51.7 mm, and the mean tree heights are 4.4, 4.1 and 4.6 m, for NT, T1 and T2 respectively, based on measurements of 45 trees for each plot immediately after the thinning treatments were applied. The crown heights range from around 1.5 m to 5 m. There was no any significant difference on the initial stand conditions (tree DBH and height) among NT, T1 and T2.

The mean annual precipitation from 1986 to 2014 was 763 mm with less than one-third precipitation in the growing season (between June to October) and the mean annual temperature is 1.9 °C. The year 2017 was classified as a drought year based on the Standardized Precipitation Index (SPI). The mean daily temperature during the growing season of 2017 is 12.1 °C, and the total growing season precipitation is 37.4 mm. Detailed information on the study site and the experimental design can be found in Wang et al. [100].

### Leaf-level measurements

Leaf-level WUE (μmol mmol^−1^) was calculated as the ratio of leaf photosynthesis rate (A, μmol CO_2_ m^−2^s^−1^) to leaf transpiration rate (T_leaf_, mmol H_2_O m^−2^s^−1^). Leaf-level WUEi (μmol mol^−1^) was calculated as the ratio of A to stomatal conductance (g_s_, mol H_2_O m^−2^s^−1^).

1 $$\text{Leaf-level}\;\text{WUE}\;\text{ = }\;\frac{\text{A}}{{\text{T}_{{\text{leaf}}} }}$$

2 $${\text{Leaf-level}}\;{\text{WUEi }} = \frac{A}{{g_{s} }}.$$

Under the steady state condition (Beer et al. [9] when the leaf temperature equals to the air temperature, and when the water vapor pressure difference between inner leaf and ambient air can be approximated by atmospheric VPD [109], the leaf-level photosynthesis (A) transpiration (T_leaf_), WUE and WUEi can be computed using Fick’s law [38]. As described by Zhou et al. [109],

3 $${\text{A}} = g_{s} \frac{{\left( {c_{a} - c_{i} } \right)}}{{1.6 p_{a} }}$$

4 $$T_{leaf} = g_{s} \frac{VPD}{{ p_{a} }}$$

5 $${\text{Leaf-level}}\; {\text{ WUE }} = \frac{A}{T_{leaf}} = \frac{{\left( {c_{a} - c_{i} } \right)}}{1.6 VPD}\text{ = }\;\frac{{c_{a} \left( {1 - \frac{{c_{i} }}{{c_{a} }}} \right)}}{1.6 VPD}$$

6 $${\text{Leaf-level}}\;{\text{WUEi }} = \;\frac{A}{{g_{s} }} = \frac{{\left( {c_{a} - c_{i} } \right)}}{1.6 } = \;\frac{{c_{a} \left( {1 - \frac{{c_{i} }}{{c_{a} }}} \right)}}{1.6}.$$

where, g_s_ is the stomatal conductance of H_2_O (μmol m^−2^s^−1^), c_a_ is the atmospheric CO_2_ concentration (μmol mol^−1^); c_i_ is the intercellular CO_2_ concentration ((μmol mol^−1^); and p_a_ is the atmospheric pressure (hPa); and the factor 1.6 arises from the fact that g_s_ is 1.6 times larger than leaf CO_2_ conductance.

According to Eq. (5) and (6), the leaf-level WUEi can be regarded as the product of leaf-level WUE and the leaf to air vapour pressure deficit (i.e., leaf-level WUEi = leaf-level WUE × VPD, or alternatively, leaf-level WUE = leaf-level WUEi/VPD) [49, 62, 90, 103].

All the variables were measured using the LI-6400XT instantaneous photosynthesis measurement system (Licor, Lincoln, NE, USA) with an opaque conifer chamber (Model 6400-22) and an external RGB light source (6400-18A). Measurements were made approximately once per week, from June, 23rd, 2017 until October 08th, 2017, between 10:00 am and 14:00 pm. On each measurement date, five trees in each plot from the same block were randomly selected, and for each tree, four bunches of needles orienting north, south, east and west at the similar location of the bottom layer of the tree canopy (around 1.5–1.7 m height) were logged for three consecutive times. We took the average of the three consecutive records for each aspect per tree. In order to mimic the natural environment when conducting the chamber measurement, the temperature was set as the ambient temperature, the light conditions were set to reflect the ambient light levels, and the leaf chamber was sealed with gum in case of gas leakage. Flow rate of air was set at 500 μmol s^−1^ to minimize any effect of the equipment on the environmental variables [74]. The microclimatic variables inside the leaf chamber sometimes might not exactly correspond to their outside environments, therefore, the outputs including temperature, VPD and PAR measured from the sensors inside the chamber were selected for analyzing leaf level responses to microclimates.

### Tree-level measurements

Tree-level WUE (mm^2^ kg^−1^) is calculated as the ratio of basal area increment (BAI, mm^2^d^−1^) to tree transpiration (T_tree_, kgd^−1^).

7 $${\text{Tree-level}} \; {\text{WUE }} = \;\frac{BAI}{{T_{tree} }}.$$

There were five trees per plot in B1 (namely sap flow trees) (15 trees in total) installed with the Granier-type sap flow probes (Model TDP-30, Dynamax, Inc., Texas, USA) at the breast height (1.3 m above ground). Tree transpiration was calculated based on the sapwood area and the continuously measured sap flow velocity as decribed by Wang et al. [100]. Detailed descriptions of DBH and sap flow measurements and their related quality control can also be found in Wang et al. [100]. BAI was calculated based on the diameter at breast height (DBH) of the five sap flow trees per plot in B1 by an electronic caliper (Model:500-196-30, Mitutoyo Corporation, Japan) at the beginning and the end of each growing season of 2016 and 2017. The DBH of 45 trees per plot across the three blocks was measured monthly using the same electronic caliper in the two growing seasons and was used in the allometric equation for calculating stand-level above-ground biomass and the stand transpiration as described in the next section.

### Stand-level estimations

Stand-level WUE (kg m^−3^) is calculated as the ratio of stand net primary production (NPP, kg m^−2^ d^−1^) to stand transpiration (T_stand_, mm d^−1^). Stand-level intrinsic WUE (stand-level WUEi, kg m^−3^) is calculated as the ratio of NPP to canopy conductance (Gs, mm d^−1^).

8 $${\text{Stand-level}}\;{\text{WUE }} = \frac{NPP}{{T_{stand} }}$$

9 $${\text{Stand-level}}\;{\text{WUEi }} = \frac{NPP}{{G_{S} }}.$$

Given that the studied stand is an even-aged and mono-specific forest with sparse understory, NPP is estimated by changes in the stand above-ground biomass (AGB_stand_, g) in each growing season, and T_stand_ (mm d^−1^) is estimated from the tree transpiration, stand density and DBH distribution, as reported in Wang et al. [100]. Tree AGB (AGB_tree_) is estimated by the tree allometric equations based on 24-year-old lodgepole pine trees from a range of stand densities across the Yellowstone subalpine plateaus [22].

10 $${\text{AGB}}_{\text{tree}} \left( {\text{g}} \right) \, = { 98}. 8 5\times {\text{basal diameter }}\left( {\text{cm}} \right)^{ 1. 9 9}$$

11 $${\text{AGB}}_{\text{stand}} = {\text{ mean AGB}}_{\text{tree}} \times {\text{ stand density}}$$

12 $${\text{NPP }} = \;{\Delta\text{AGB}}_{\text{stand}}.$$

Paired measurements of tree DBH and basal diameter from 180 trees across all three blocks were used to build a linear relationship between basal diameter and tree DBH, as reported in our prevous study [100].

Gs was used as a proxy to indicate stomatal response at the canopy level. It was calculated by the simplified inversion of the Penman–Monteith equation, assuming that the VPD is close to the leaf to air vapor pressure deficit with no vertical gradient through canopy, and negligible water storage above the point where sap flow probes were inserted [34]. This method has also been applied in lodgepole pine forests [89]. The conditions of our young lodgepole pine stands, including low canopy height (< 2.5 m) and relatively open canopies (canopy closure < 55%), generally satisfy the assumptions of the equation.

13 $${\text{Gs}} = \frac{{\gamma \lambda E_{LA} }}{{\rho_{a} c_{a} VPD}}$$

where, ƴ is the psychrometric constant (0.067 kPa K^−1^); λ is the latent heat of vaporization calculated by Harrison’s equation, $\lambda \; = ;2.501\;{ -}\;2.361\; \times \;10^{{{-}3}} \;T_{a}$ [55]; E_LA_ is the transpiration per leaf area, E/LA (mms^−1^); ρ_a_ is air density (1.225 kg m^−3^); c_a_ is the specific heat of air (1.0 × 10^−3^ MJ kg^−1^ K^−1^); and VPD is vapor pressure deficit (kPa). All VPD data used in the equation are greater than 0.6 kPa to minimize the relative errors (< 10%) [34].

Monthly leaf area (LA, m^2^) was also estimated from the tree allometric equations from Copenhaver and Tinker [22].

14 $$\text{LA}\;\left( {\text{in m}^{{2}} } \right)\; = 0.02{ \times }\;\text{Basal diameter}\left( {\text{in cm}} \right)^{2.34}$$

Leaf area index (LAI) was estimated by dividing LA by the projection coefficient (2.5) [64]. The estimated mean monthly LAI in 2016 for C (0.96) matches relatively well with the field measurement (0.97).

### Collection of climate data

Climatic variables including solar radiation (Rn, W m^−2^), air relative humidity (RH,  %), temperature (T, °C), precipitation (P, mm) and wind velocity (Wv, m s^−1^) were continuously measured in each treatment in B1 by a HOBO weather station (Onset Computer, Bourne MA, USA). The sensors were placed at canopy level (approximately 2.5 m). VPD is calculated based on Goff–Gratch equation [48]. Microclimate variables including leaf temperature, leaf VPD and incoming photosynthetically active radiation (PAR, µmol (photons) m^−2^ s^−1^), at a height of approximately 1.5–1.7 m, across the three blocks (9 plots) at mid-day on a weekly basis during the growing season, were recorded by the instantaneous photosynthesis measurement system (Model LI-6400XT, Licor, Lincoln, NE, USA). Soil volumetric water content (VWC) at two depths (20 and 40 cm) in three randomly selected locations per plot in B1 was measured by EC-5 sensors (Decagon, Pullman, WA, USA) at 20-minute intervals. Soil VWC at the 20 cm depth was also manually measured weekly in the three blocks using a GS-1 portable measuring system (Decagon, Pullman, WA, USA).

### Statistical analysis

The instantenous leaf-level WUE and WUEi were first analysed by multi-factor AVOVA to investigate the effects of branch aspect, thinning, date and their interactions. Since there were no significant effects of the branch aspect and its interactions (Additional file 1: Table S2), measurements of the four aspects per tree were averaged to yield more integral responses of leaf-level WUE and WUEi for each tree. The averaged leaf-level WUE and WUEi per tree were analyzed by the two-way ANOVA to investigate the effects of thinning, date and their interactions (5 replicates per measuring date for each treatment). Tree-level WUE, BAI and transpiration of the 15 monitoring trees during the two growing seasons were analyzed by ANCOVA with the initial DBH of the trees as covariate, and the thinning treatments and the year as factors (5 replicates per treatment per growing season). Stand-level WUE values were calculated for each plot and then were analyzed by the two-way ANOVA analysis with the thinning treatments and the year as factors (3 replicates per treatment per growing season). Stand-level WUEi were only anlyszed in 2017 due to the requirement of the canopy conductance model on that VPD should be greater than 0.6 kPa (3 replicates per treatment). Model residuals were checked to meet the requirements of normality and homoscedasticity of variance. In most cases, the assumptions were satisfied, except the stand-level WUE and NPP, even though multiple data transformation methods including log, square roots and cubic roots and Box-Cox transformation were applied. Therefore, comparisons on stand-level WUE and NPP between two groups of the treatments or between years were performed with independent-t test, if data met the requirements of homogeneity of variance and normality, or alternatively, the Mann–Whitney U test if those assumptions were violated.

In order to analyze the responses of tree-level WUE to microclimatic variables, the monthly tree-level WUE for each tree was calculated as the ratio of the monthly increment of the tree BAI to the corresponding tree transpiration during the same period. These monthly tree-level WUE were further grouped to yield the average monthly tree-level WUE under each thinning treatment. The responses of the averaged monthly tree-level WUE for NT, T1, and T2 to their corresponding mean daily values of microclimatic variables under the same time interval were analyzed by Spearman correlation test. If the results of the correlation test were significant, the relationship between the monthly tree-level WUE for NT, T1 and T2 with microclimate were fitted by the establishd equations as summarized in the Additional file 1: Table S1. The responses of the monthly stand-level WUE and WUEi under each treatment (i.e., the average of the three plots under the same thining treatment) to corresponding microclimate were analyzed in the similar way as the tree-level analyses. It is note that DBH data were subject to some measuring errors, which resulted in the negative increments of tree BAI. These negative values were keep in the analyses for the responses of WUE and WUEi to the thining treatments of the two growing seasons. However, these negative valudes were excluded in the analyses for WUE–or WUEi -microclimate relationships, as they would be unrealistic for microclimate variables to correspond with negative WUE or WUEi values. Monthly stand-level WUEi were only available in August of 2016 and in July and August of 2017 when VPD conditions were appropriate for the application of the simplified Penman–Monteith equation introducd in the previous section. A significance level of p < 0.05 was used for all analyses. All data were processed by R (R Core Team (2014) and SPSS for Windows (SPSS, Inc., USA).

## Results

### Effects of thinning on leaf-level WUE and WUEi

**Fig. 3:**
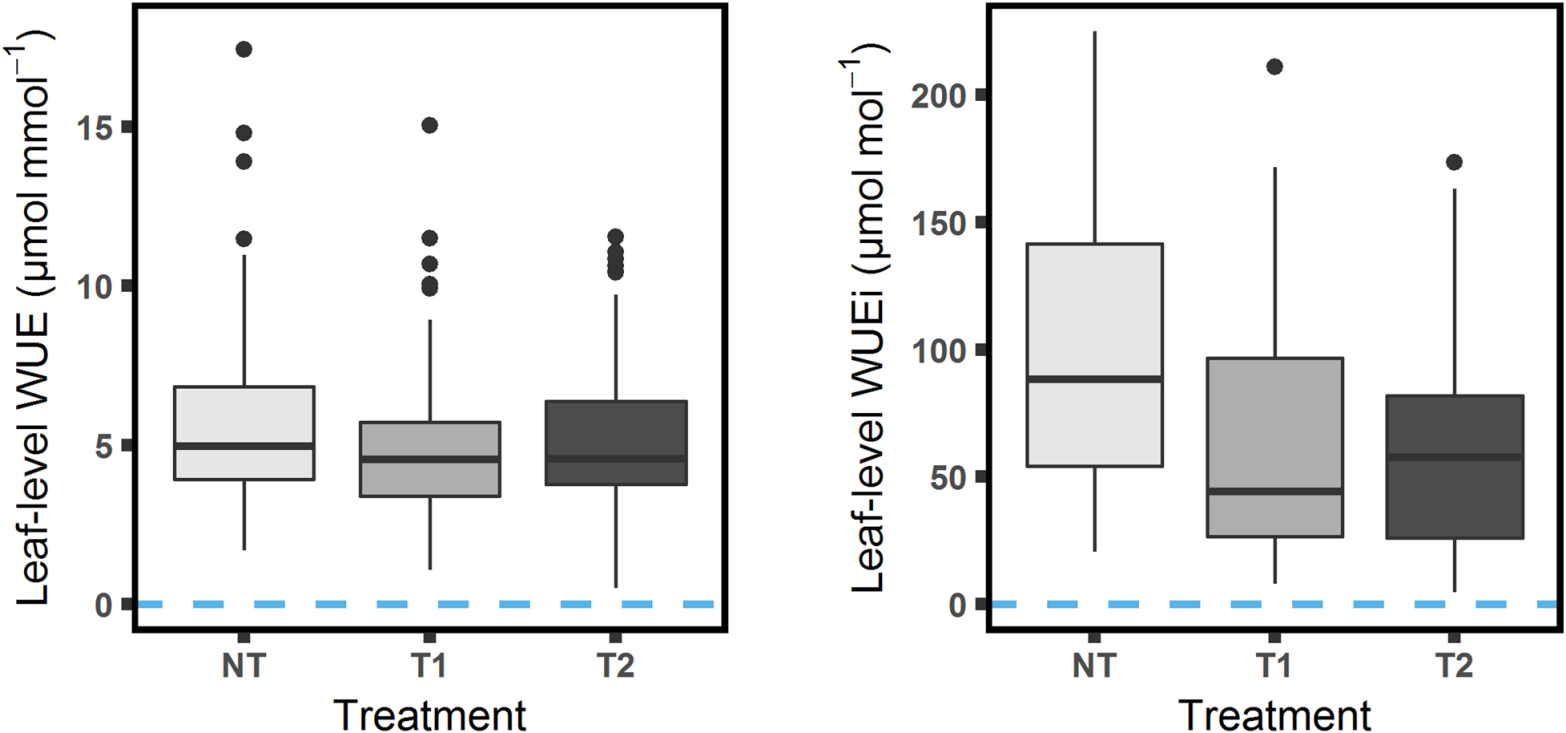
Leaf-level WUE and WUEi during the growing season of 2017

Thinning did not significantly affect leaf-level WUE (p = 0.65, Additional file 1: Table S3), with mean leaf-level WUE values for NT, T1 and T2 being 5.91 ± 3.25, 5.63 ± 4.65 and 5.30 ± 2.76 μmol mmol^−1^, respectively. However, thinning had an sigfnisicant impact on leaf-level WUEi (p < 0.001, Additional file 1: Table S3), with the averaged leaf-level WUEi for NT, T1 and T2 being 98.41 ± 52.89, 64.23 ± 50.39 and 61.82 ± 40.99 μmol mol^−1^, respectively. Leaf-level WUEi in NT was statistically higher than those in T1 and T2 (both p < 0.001), while T1 and T2 did not significantly differ from each other (p = 0.80) (Fig. 3).

### Effects of thinning on tree-level WUE

The ANCOVA test showed that thinning significantly affected tree-level WUE (p = 0.026, Additional file 1: Table S4). Looking into the separate growing seasons, the mean tree-level WUE for NT, T1 and T2 in 2016 were 0.09 ± 0.64, 0.85 ± 1.27 and 0.81 ± 0.90 mm^2^ kg^−1^, respectively, and they were not significantly different from each other (p = 0.403). These values were reduced to 0.08 ± 0.31, 0.39 ± 0.18 and 0.49 ± 0.18 mm^2^ kg^−1^ in 2017, respectively, and only tree-level WUE in NT and T2 were statistically different (p = 0.03). Thus, the heavier thinning significantly improved tree-level WUE in the drought year.

**Fig. 4:**
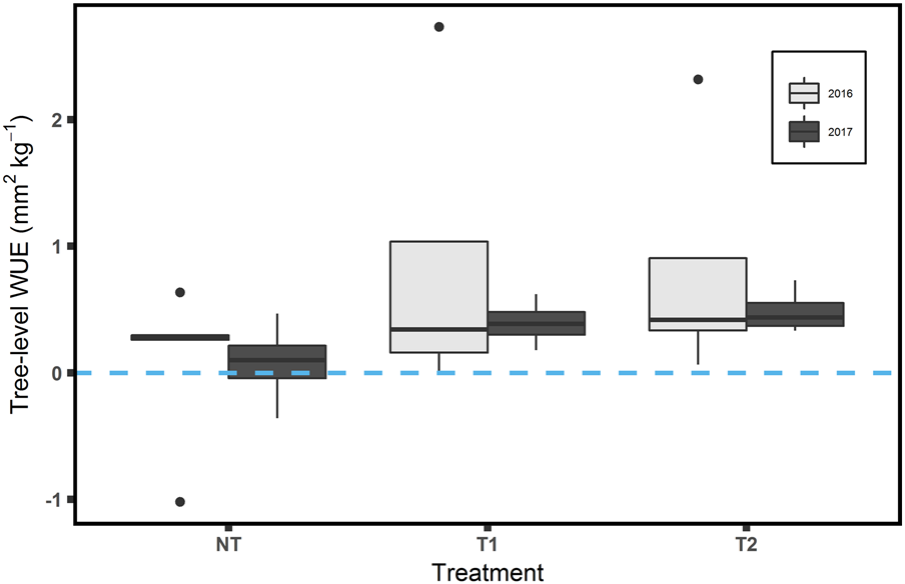
Tree-level WUE during the growing season of 2016 and 2017

However, the ANCOVA test showed that tree-level WUE did not differ between years for the three groups (p = 0.56, Additional file 1: Table S4). This was probably due to the large variances in the tree-level WUE of T1 and T2 in the non-drought year (Fig. 4). In addition, there was no significant interaction effect between the year and thinning (p = 0.84, Additional file 1: Table S4).

### Effects of thinning on the stand-level WUE and WUEi

Thinning did not have significant impacts on the stand-level WUE by pooling two-year data together (one-way ANOVA, p = 0.18). In 2016, the stand-level WUE values were 0.05 ± 0.85, 0.62 ± 0.55, and 0.63 ± 0.69 kg m^−3^ for NT, T1 and T2, respectively, and there were no significant differences among them (one-way ANOVA, p = 0.55). In 2017, the stand-level WUE values were changed to 0.31 ± 0.08, 0.56 ± 0.11, and 0.70 ± 0.13 kg m^−3^ for NT, T1, and T2, respectively, with the WUE in NT being significantly lower than those in T1 (p = 0.03) and T2 (p = 0.005), suggesting the positive thinning effects only occurred in the drought year. However, there was no statistical difference between T1 and T2 (p = 0.18). Besides, when comparing stand-level WUE between years for each group of NT, T1 and T2, no significant differences were found (all p > 0.1).

**Fig. 5:**
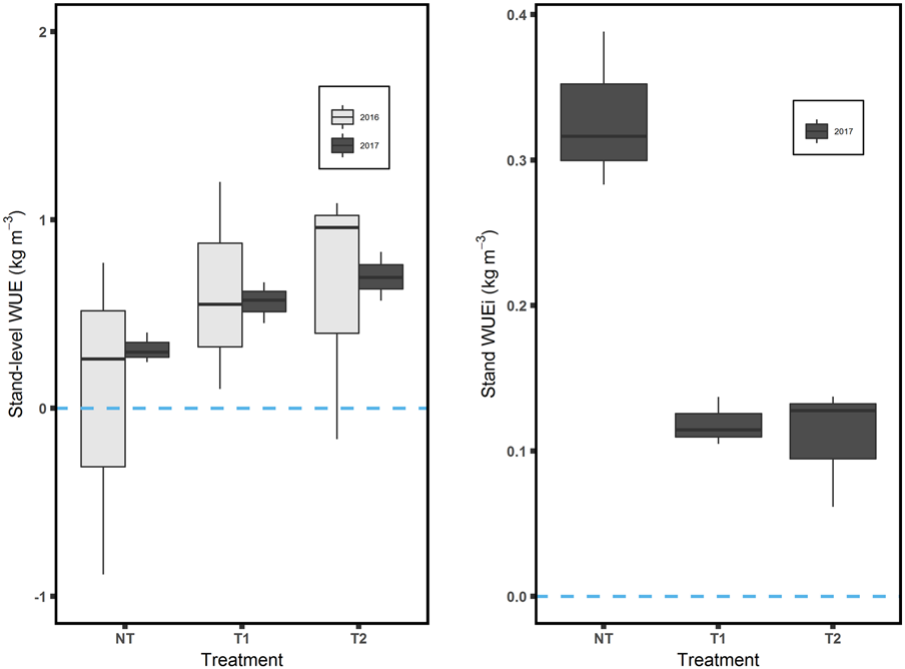
Stand-level WUE in 2016 and 2017, and stand-level WUEi in 2017

Stand-level WUEi values in 2017 were 0.36 ± 0.06, 0.13 ± 0.02, and 0.12 ± 0.04 kg m^−3^ for NT, T1 and T2 respectively. NT had statistically higher WUEi than T1 and T2 did (both p = 0.001), and there was no significant difference between T1 and T2 (p = 0.77) (Fig. 5).

### Responses of leaf-level WUE and WUEi to VPD and PAR

**Fig. 6:**
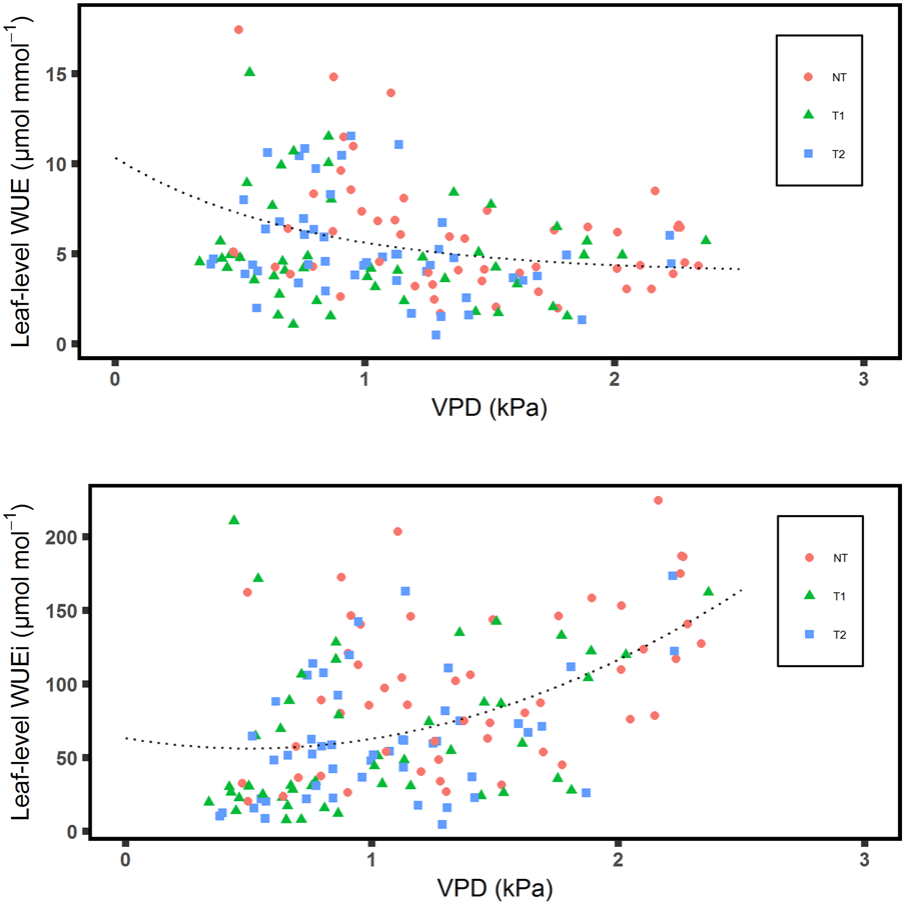
Leaf-level WUE and WUEi versus VPD for NT, T1 and T2. The top panel shows the leaf-level WUE versus VPD with a fitted exponential decay equation (leaf-level WUE = 3.92 + 6.416e^−1.325VPD^ (R^2^ = 0.074). The bottom panel shows the leaf-level WUEi versus VPD with a fitted parabolic equations (leaf-level WUEi = 27.08VPD^2^-27.45VPD + 63.32 (R^2^ = 0.23)

**Fig. 7:**
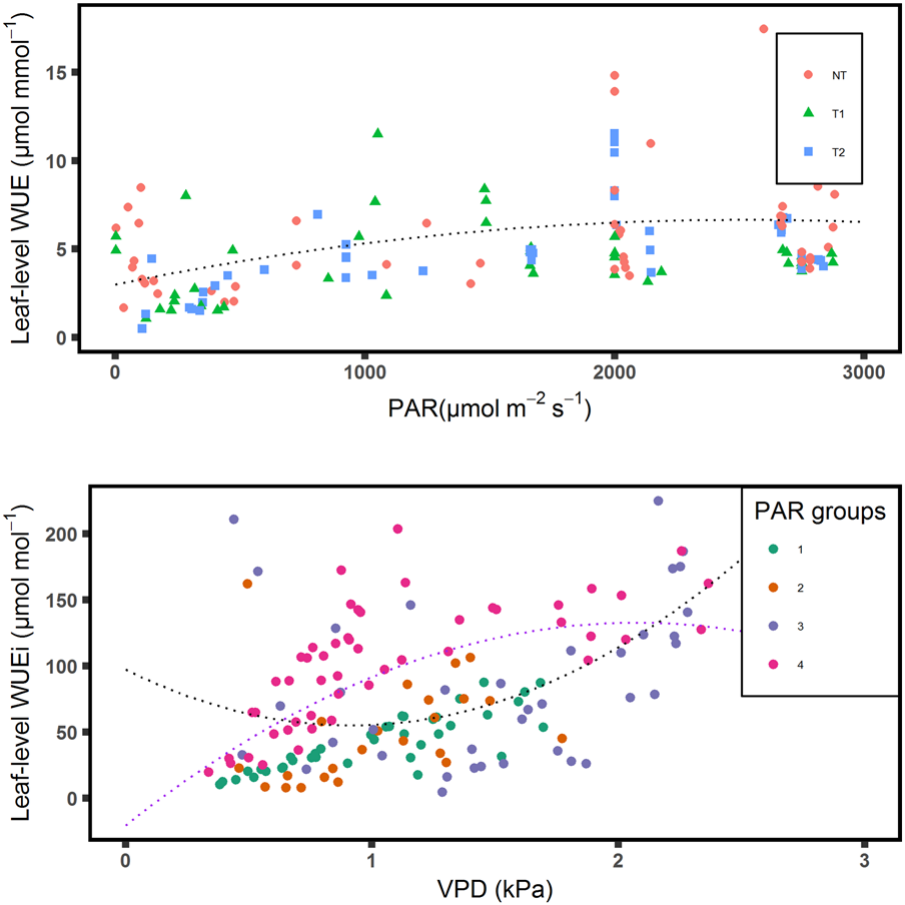
Leaf-level WUE versus PAR (upper) and leaf-level WUEi versus VPD for PAR groups (PAR in μmol/m^2^s) (group 1: PAR <=500; group 2: PAR > 500 and <=1500; group 3: PAR > 1500 and <= 1500; and group 4: PAR > 2500). The top panel shows the leaf-level WUE versus PAR with a fitted parabolic equation (leaf-level WUE = -5.74 × 10^−7^PAR^2^ + 2.90 × 10^−3^PAR + 2.99 (R^2^ = 0.13). The bottom panel shows the leaf-level WUEi versus VPD for the four PAR groups with fitted parabolic equations (when PAR < = 2500: leaf-level WUEi = 50.28VPD^2^-92.19VPD + 97.44 (R^2^ = 0.27) and when PAR > 2500: leaf-level WUEi = -35.59VPD^2^ + 147.73VPD-20.45 (R^2^ = 0.37)

Leaf-level WUE was significantly negatively correlated with VPD (spearman rho = -0.25, p = 0.002, Table 1), while leaf-level WUEi exhibited a significantly positive relationship with VPD (rho = 0.45, p < 0.001, Table 1). Both the responses of leaf-level WUE and WUEi to VPD did not significantly differ among NT, T1 and T2, but the VPD relationship with leaf-level WUE was best fiting with the exponential decay equation (R^2^ = 0.074, Additional file 1: Table S1), while that with leaf-level WUEi was best with the parabolic equation (R^2^ = 0.23, Additional file 1: Table S1) (Fig. 6). As for their responses to PAR, only leaf-level WUE exhibited significant correlations (rho = 0.33, p < 0.001, Table 1), and there were no significant differences among NT, T1, and T2 (Fig. 7). However, the responses of leaf-level WUEi to VPD were different when PAR was higher than 2500 μmol/m^2^s (Fig. 7).

**Table 1:** Correlation coefficients between WUE with VPD, light intensity and soil water content

WUE across spatial scales	VPD	Light intensity (PAR or Transmitted solar radiation)	Soil water content at 20 cm	Soil water content at 40 cm
Leaf-level				
Leaf-level WUE	− 0.25***	0.33***	− 0.25	− 0.37**
Leaf-level WUEi	0.45***	0.11	− 0.47***	− 0.55***
Tree-level				
Tree-level WUE	− 0.54**	− 0.37	0.07	− 0.03
Stand-level				
Stand-level WUE	− 0.79***	− 0.61**	− 0.005	− 0.14
Stand-level WUEi	− 0.20	− 0.85***	− 0.40	− 0.64*

Light intensity includes PAR at the leaf level and transmitted solar radiation at tree and stand levels. Star indicates the significant level at 0.01***, 0.05**, and 0.1*

### Responses of tree-level WUE to VPD and transmited solar radiation

**Fig. 8:**
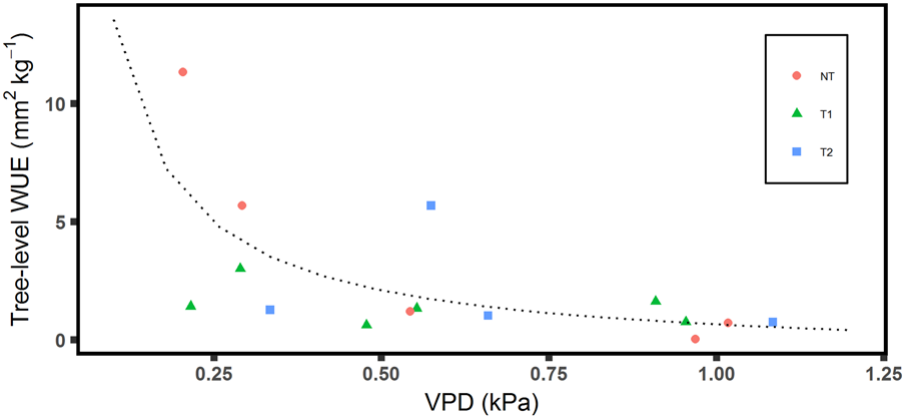
Tree-level WUE versus VPD with a fitted hypobolar equation (Tree-level WUE = 1.43/VPD +-0.77 (R^2^ = 0.41))

Tree-level WUE was negatively and significantly correlated with VPD (rho = −0.54, p = 0.04, Table 1), and there were no any significant differences among NT, T1 and T2 (Fig. 8). There were no significant correlations between tree-level WUE and transmitted solar radiation (p = 0.18), and the transmited solar radiation did not significantly influence the responses of tree-level WUE to VPD.

### Responses of the stand-level WUE and WUEi to VPD and transimited solar radiation

**Fig. 9:**
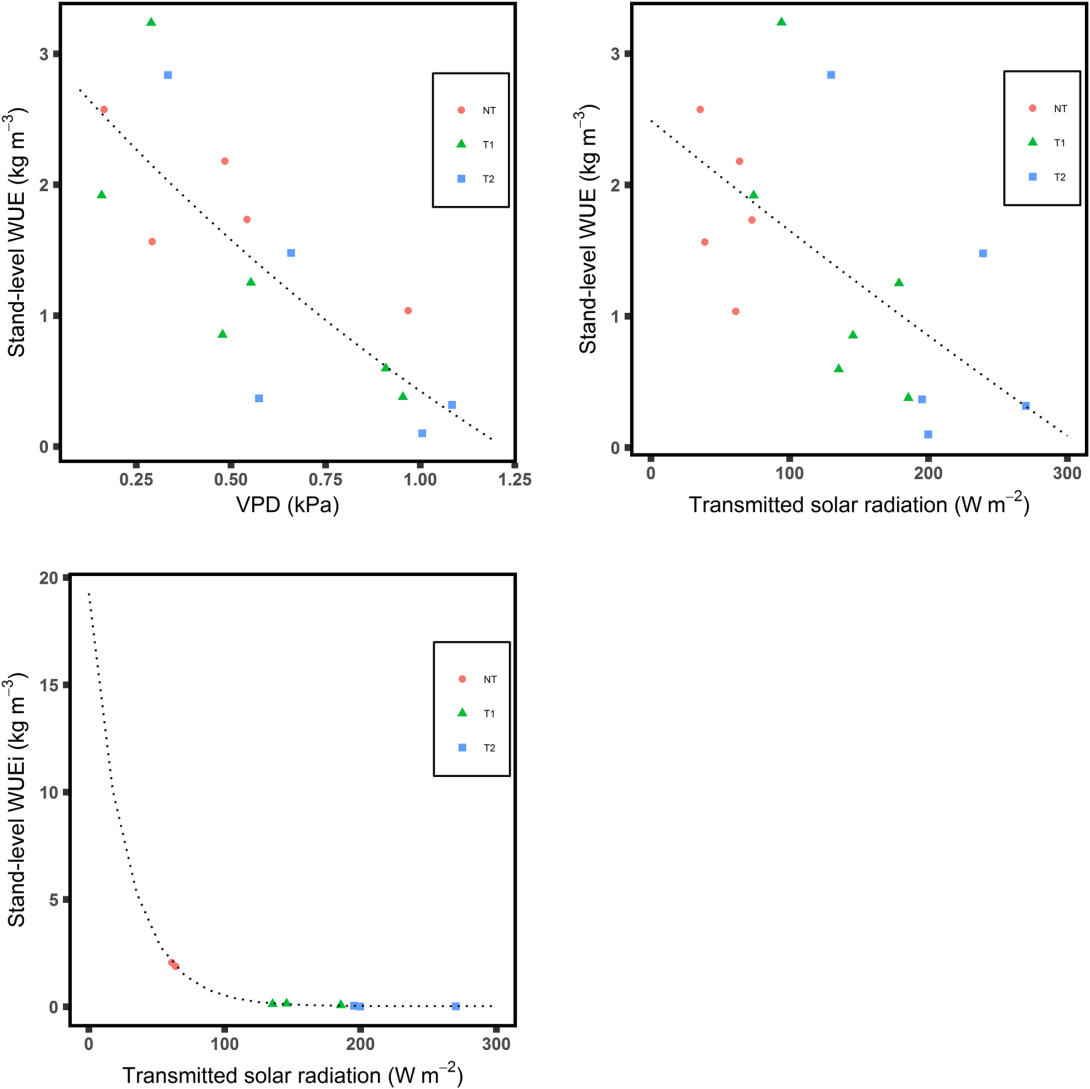
Stand-level WUE versus VPD (stand-level WUE = 3.78 + 6.83e^−0.49VPD^ (R^2^ = 0.63), and stand-level WUE and WUEi versus transmitted solar radiation for NT, T1 and T2 (stand-level WUE = 17.87e^−0.00048×transmitted solar radiation^ -15.38 (R^2^ = 0.37); And stand-level WUEi = 19.24e^−0.037×transmitted solar radiation^ +0.025 (R^2^ = 1))

The stand-level WUE was negatively and statistically correlated with VPD (rho = −0.79, p < 0.001, Table 1) and transmited solar radiation (rho = −0.61, p = 0.01, Table 1), but the correlations with VPD and with transmitted solar radiation did not significantly differ among NT, T1 and T2 (Fig. 9). However, there was no any significant correlation between stand-level WUEi and VPD (p = 0.64), but stand-level WUEi was significantly correlated with transmitted solar radiation (rho = −0.85, p = 0.008) (Fig.  9).

## Discussion

### The effects of thinning on WUE across the spatial scales

In contrast to our first hypothesis, leaf-level WUE values were not significantly different among NT, T1 and T2, while leaf-level WUEi was statistically higher in NT than in the thinned stands, and there were no significant differences between the two thinning intensities. Comparing the underlying processes of leaf-level WUE and WUEi, leaf photosynthesis, transpiration and stomatal conductance were all significantly lower in NT than in the thinned stands (all p < 0.001), with no statistical difference between T1 and T2 (all p > 0.1) (Additional file 1: Figure S1), which is in accordance with previous studies showing higher leaf photosynthesis, leaf transpiration and stomatal conductance in the lower density stands with a wide range of tree densites and species [12, 80, 97]. Therefore, similar leaf-level WUE between the treatments in our study can be attributed to similar enhancements of the leaf photosynthesis and transpiration, while the higher leaf-level WUEi in NT might be due to more positive effects of thinning on stomatal conductance than on the leaf transpiration.

Our results for leaf-level WUEi between T1 and T2 agrees with studies showing that thinning has no effects on WUEi in Scots pine (*Pinus Sylvestris)* and maritime pine (*Pinus pinaster)* [81], Aleppo pine (*Pinus halepensis* Mill.) [36], and black pine (*Pinus nigra* Arn.) [69] stands in Mediterranean forests, based on the isotope method. However, our results are in contrast to studies which were conducted using the leaf gas-exchange measurements in young paper birch (*Betula papyrifera* Marsh.) stands [99] and in oak saplings (*Quercus cerris* L.), and in ash (*Fraxinus ornus* L.) forests [24]. They attributed the increased leaf-level WUEi to increased PAR induced by their thinning treatments [24, 99].

In our study, VPD, instead of PAR, might explain the discrepancy between the responses of leaf-level WUE and WUEi, and the significantly higher leaf-level WUEi in NT. VPD during the leaf measurement periods was statistically higher in NT than in T1 and T2 (both p < 0.001) with no significant differences between T1 and T2 (p = 0.29, Additional file 1: Figure S4) and leaf-level WUEi approximates the product of leaf-level WUE and VPD [62, 103]. Further, in comparison with temperature and PAR, VPD was the only microclimate variable that significantly differed in the control than in the thinned stands during the leaf measurement periods. As VPD is determined by temperature and RH, and RH is influenced by soil evaporation and plant transpiration [5, 25, 39], higher VPD in NT can be attributed to a lower soil water content and a higher stand transpiration in NT, as shown in our previous study [100]. VPD can indicate that atmospheric drought [14], and increased WUEi are commonly observed under drought conditions (Andrés et al. [4, 36, 61]. Therefore, higher leaf-level WUEi in NT suggests that the unthinned stand experienced more severe water stress than the thinned stands.

However, VPD could either increase [5, 51, 101], or remain unchanged [18, 76, 78, 86] with increasing thinning intensities. Though microclimates play important roles in influencing leaf-level WUEi, their effects may be site-specific. We also acknowledge that the field measurements in our study were conducted from 10 am to 14 pm, and consequently, the results may not fully represent the effects of thinning on mean daily leaf-level WUE and WUEi on a 24-h basis. This shortcoming could be addressed by using the isotopic method, which will be considered in our future study.

At the tree level, we found significant and positive impacts of thinning on tree-level WUE (Fig. 4, Additional file 1: Table S4), tree BAI and tree transpiration (Additional file 1: Table S4 and Additional file 1: Figure S2). Our result agrees with other studies showing that thinning increased tree-level WUE in Aleppo pine (*Pinus halepensis* Mill.), Silvertop (*Eucalyptus nitens)* (Deane and Maiden) and Norway spruce (*Picea abies* [L.] *Karst.)* forests [36, 40, 44], a conclusion based on observations of enhanced tree transpiration accompanied with increased tree growth. Park et al. [83] found that the significant differences in tree-level WUE between heavy thinning and control stands only occurred in high growth years in a 50-year-old Korean pine (*Pinus koraiensis)* stand. The enhancement of tree-level WUE was mainly attributed to a lower water stress under a more intense thinning treatment [15, 27, 44, 83]. Therefore, the effects of thinning on tree-level WUE depend on how thinning reduces tree competition for resources (e.g., water, light and nutrients) as indicated by Fernández-de-Uña et al. [37], and thus these can be more pronounced in a drought year, as observed by Park et al. [83]. This has been confirmed by our study in which increasing tree-level WUE associated with a higher thinning intensity was more obvious in the drought year (2017) than in the normal year (2016).

The discrepancy of WUE between the tree and leaf levels in 2017 suggested that the responses of tree-level WUE to thinning treatments is more resulted from tree-level physiological processes instead of the leaf-level changes. Tree-level physiological changes due to thinning treatments may include the stem respiration processes [75], the tree carbon allocation pattern under droughts [87], and the night-time transpiration [33, 75]. Stem respiration, however, was greater in the thinned stands [59], and thus is unlikely to contribute to a possible explanation of our observations. Under water stress, lodgepole pine tends to allocate more biomass to their root system to improve water acquisition [87], which may serve as a potential explanation. In order to infer the effect of the night-time transpiration, we calculated the percentage of the tree daily night sap flow (from 1 h after sunset to 1 h before dawn [75] to the tree daily total sap flow, since the night sap flow is usually partitioned into the night transpiration and the stem refilling [41]. We found that tree daily night sap flow accounted for 29.7 ± 26.1%, 11.9 ± 14.1% and 6.5 ± 11.9% of the total tree daily sap flow in 2017 for NT, T1 and T2, respectively, all of which were significantly different from each other (all p < 0.001). If tree transpiration did occur at night at our study site during the experiment period, it is possible that the trees in NT had the highest daily non-productive water consumption, while those in T2 had the lowest, contributing to a significantly low tree-level WUE in NT but high tree-level WUE in T2. The fact that night transpiration reduces whole-plant WUE and consequently causes a lack of correspondences in WUE between leaf and whole-plant scales has been well documented [33, 75]. But in our study, the night-time VPD was generally highest in T2, followed by NT, and then T1 (Additional file 1: Figure S5), and soil water contents were substantially higher in the thinned stands [100], so that VPD and soil water contents alone can not explain the patterns of tree night sap flow found in our study. However, under drought conditions, stomatal conductance can be unrelated to VPD and soil water content, which leads unavoidable water loss through the epidermis of tree needles (e.g., 6−8% of daily transpiration under well-watered conditions in comparison with 19−20% of daily transpiration during drought) [16]. This may help to explain the result of our study. Nevetheless, our conclusion was only based on one drought year. Continuous monitoring of the study site is needed to further strengthen the mechnismes behind the thinning and drought effects.

Stand-level WUE responses to the thinning treatments were slightly different from the tree-level WUE in this study. There was the lack of significance of the overall thinning effects on the stand-level WUE in the normal year of 2016, suggesting that forest structural properties damped the WUE responses to the thinning treatments from the tree to stand levels. It is however, also possible that the large variations in stand-level WUE in 2016 might obscure the effects of thinning (Fig. 5).

Our stand-level WUE result is within the range of the AGB-based WUE [40], and agree well with studies reporting that thinning or drought increased stand-level WUE [40, 91]. In fact, stand-level or ecosystem WUE can either increase [91], remain unchanged [91] or even decrease (Gao et al. [43] under drought conditions, depending on forest characteristics (e.g., mixed or monospecific), tree species and environmental conditions. The increased stand-level WUE in the drought year in our study were likely attributed to slightly decreases in the net accumulation of stand aboveground biomass acommpanied by a greater reduction in stand transpiration under the drought (Additional file 1: Figure S3).

Surprisingly, the stand-level WUEi was significantly higher in NT than those in the thinned stands with no significant difference between T1 and T2. The net accumulation of stand above-ground biomass was significantly higher in NT than those in T1 (p = 0.004) and T2 (p = 0.001), and there was no significant difference between T1 and T2 (p = 0.11). Canopy conductances, however, were not significantly different among the three groups (all p > 0.1, except for the comparision between T1 and T2 (p = 0.053)). Therefore, the pattern of the stand-level WUEi in our study was mainly driven by the net stand above-ground biomass accumulation during the growing season. This indicates that the higher stand density in NT compensated for the decreased individual tree growth, leading to a higher net stand above-ground biomass accumulation in the unthinned stands than those in the thinned ones. Although T2 had the highest individual tree growth, the low density in T2 counterbalanced the improved individual tree growth under the thinning treatment.

Responses of WUE and WUEi to microclimate under the thinning treatments and the implications for upscaling and modelling.

The responses of WUE from leaf to stand levels to VPD in our study agree with previous research on various types of forest ecosystems in various climatic zones [58] including beech (*Fagus sylvatica* L.) [62] oak-hickory (*Quercus and Carya* spp.) [7], sugar maple (*Acer saccharum)*, hemlock (*Tsuga canadensis)*, yellow birch (*Betula alleghaniesis)*, basswood (*Tilia americana)*, and American hophornbeam (*Ostrya virginiana)* (Tang et al. [93], basket willow (*Salix viminalis* L.) [63] and the Norway spruce (*Picea abies* L. Karst) [79]. And the response of WUEi to VPD at the leaf level in our study is in accordance with the theoratical relationship described by multiple leaf-level models (Additional file 1: Table S1). Besides, our results also agree with the research reporting that tree-level WUE was primarily a function of VPD (Table 1) [10, 63, 83]. Moreover, we found that all the WUE from leaf to stand scales depended primarily on VPD (Table 1) and that no obvious changes in the responses of WUE and WUEi to microclimate between the treatments were detected at any studied spatial scale, which supports the result from Dye et al. [31] that the relationship between WUE and VPD could be used to predict WUE in the absence of adequate background knowledge and with a limited amount of field data. However, this implication still requires further examinations with a greater sampling size and at various temporal scales, given that some minor changes in the sensitivity of WUE and WUEi to microclimates might not be fully captured in our study as Chao-Yang et al. (2018) showed that the correlations between WUE and microclimates were influenced by temporal scales and the WUE-microclimate relationships in our study were calculated based on the monthly time step alone.

The discrepancy between the leaf-level WUE and WUEi due to microclimatic factors in this study was a result of the distinct sensitivity of leaf transpiration and stomatal conductance to VPD, as leaf transpiration is regulated by both stomatal conductance and boundary layer conductance of water vapor [26], the former of which depends on the density, size and degree of opening of stomata, while the latter is determined by the air movement and leaf morphology [70]. Unlike stand-level WUE, stand-level WUEi did not signicantly correlate with VPD, which is probably because that canopy conductance in our study represented the maximum water loss from the forest stand driven by the available energy. The different responses of the WUE and WUEi at the leaf and stand scales to VPD suggest that caution must be taken in selecting a proper WUE metric for upscaling from leaf to stand levels.

Last but not least, even if the thinning did not affect the sensitivity of WUE and WUEi to microclimates, the changes in microclimate resulting from the thinning could lead to the differences in WUE between the control and thinned stands (e.g., leaf-level WUEi), and consequently affect the model prediction. Clearly, microclimate is critical in evaluating the effects of thinning from the perspective of carbon and water coupling. It also plays an important role in other ecological functions of forests, such as seed germination [30, 98, 106], species diversity [29, 47], soil nutrient cycling [21, 50, 77, 110], microhabitats for insects and animal [18, 19, 76, 88] as well as wildfire [11]; Whitehead et al. [102] and mountain pine beetle attacks [2, 8]. Previous research suggested that changes in microclimate under forest management are predictable, as microclimate is closely related to vegetation structure, elevation and microtopography [5]; Frey et al., [42, 54, 67]. It is very important for forest silvicultural practices to create suitable microclimate conditions to improve forest ecological services. Our study provides the evidence of the short-term effects of thinning on WUE from leaf to stand levels. As juvenile thinning enhances crown and rooting system development and fast growing understory vegetation [6, 11, 18, 32, 92, 96], their effects are likely dynamic, and the long-term implications of thinning require continued invenstigation and monitoring.

## Conclusions

Our study provided direct field evidence regarding the responses of WUE and WUEi to juvenile thinning treatments during non-drought and drought conditions at the various spatial scales. We conclude that: (1) the thinning treatments did not cause significant changes in all studied WUE metrics under a normal climate condition; (2) the thinning treatments, under the drought conditions, significantly increased tree-level and stand-level WUE, caused no changes in leaf-level WUE, while decreasing leaf-level and stand-level WUEi. Thus, WUE and WUEi responded differently to the thinning treatments and the drought effects at the same spatial level as well as across the different spatial scales, suggesting the importance of selecting metrics and scales when evaluating or modelling the effects of thinning on WUE; (3) no changes in the sensitivites of WUE to VPD under the thinning treatments at any studied spatial levels were detected, suggesting that the relationship between WUE and VPD may be used across the different spatial scales; and (4) only under the drought condition, the thinning significantly improved the tree- and stand-level WUE, indicating that thinning can promote forest resilience to the drought effects.

## Supplementary information

**Additional file 1:** Figures and Tables.

**Additional file 2:** Tree level, stand level, leaf level and manuscript CBM data.

## Data Availability

The data supporting this research are included within the article and its additional files (Additional file 2). Additional data are available upon request to corresponding author.
