# Supplementary material for: Responses of forest carbon and water coupling to thinning treatments from leaf to stand scales in a young montane pine forest
Source: Carbon Balance Manag. 2020 Nov 3;15:24. doi: 10.1186/s13021-020-00159-y (PMC7609426; doi:10.1186/s13021-020-00159-y)
Supplement: Supplementary file 1 — Additional file 1: Figures and Tables. [file 13021_2020_159_MOESM1_ESM.docx]

Additional file

**Theoretical responses of WUE and WUEi at the multiple spatial scales to microclimate variables (VPD, and light availability)**

VPD and light availability have been considered as the predominant factors affecting WUE, and thus were explicitly examined in this study.

According to the equations (13) and (14) in the manuscript, the relationship between leaf-level WUE and WUEi and VPD can be investigated using the ratio of intercellular CO2 concentration to the atmospheric CO2 concentration ( $\frac{c_{i}}{c_{a}}$ ) as a surrogate, since $\frac{c_{i}}{c_{a}}$ can be modelled by VPD with several different methods that were proposed by Lloyd and Farquhar (1994), Wong and Dunin (1987) and Leuning (1995). Therefore, the responses of leaf-level WUE and WUEi to VPD were fitted with VPD in the models when they were significantly correlated to VPD. The exponential decay equation between WUE and VPD at leaf and stand levels, and the reciprocal equation between WUE and VPD at individual tree and stand levels, which were adopted by previous studies (Baldocchi et al., 1987; Linderson et al., 2012; Lindroth and Cienciala, 1996; Tang et al., 2006), were also investigated in our study. The model equations and their goodness of fit (R^2^) were summarized in the Table S1, and the ones that fitted the best were selected in our study. However, no models that directly predict WUEi at the individual and stand levels by VPD are available, and consequently, we fitted the equation that yielded the highest R^2^.

Leaf-level WUEi was also dependent on PAR (increasing with the increasing PAR until the threshold was met and then remains nearly constant), and such dependence was fitted with an exponential equation (Linderson et al., 2012). Besides, the level of PAR can affect the responses of leaf-level WUEi to VPD (Linderson et al., 2012). At the ecosystem level, Tang et al. (2006) showed significant exponential relationship between ecosystem WUE and PAR in a hardwood-dominated forest. Therefore, we examined the dependence of WUE and WUEi on light availability at the multiple spatial scales by exponential relationships.

| **Model** | **Formulation/equation** | **Relationship with VPD** | | **Fitted equation (WUE)** | **Fitted equation (WUEi)** |
| --- | --- | --- | --- | --- | --- |
|  |  | **WUE** | **WUEi** |  |  |
| Leaf level | | | | | |
| Lloyd and Farquhar (1994) | $\frac{c_{i}}{c_{a}}= 1-\sqrt{\frac{1.6 VPD {(c}_{a}-\Gamma)}{\lambda_{cf}{c_{a}}^{2}}}$  Where, Γ is the leaf CO_2_ compensation point; λ_cf_ is the ratio of ∂T_leaf_/∂g_s_ to ∂A/∂g_s_ ,which is almost constant for certain vegetation in the short term. | $\propto\frac{1}{\sqrt{VPD}}$ | $\propto\sqrt{VPD}$ | = 3.90/$\sqrt{VPD}$+1.68  (R^2^=0.070 ,p=0.001) | =91.304$\sqrt{VPD}$-20.35  (R^2^=0.19, p<0.001) |
| Wong and Dunin (1987) | $\frac{c_{i}}{c_{a}}= 0.96-0.0194VPD+3.282\times{10}^{-4}{VPD}^{2}$ | =aVPD+b/VPD+c | =aVPD^2^+bVPD+c | =-0.632VPD+1.182/VPD+5.082  (R^2^=0.068, p=0.002) | =27.08VPD^2^-27.45VPD+63.32  (R^2^=0.23, p<0.001) |
| The Ball-Berry model revised by Leuning (1995) | $\frac{c_{i}}{c_{a}}= 1-\frac{1-\frac{\Gamma}{c_{a}}}{m_{L}}(1+\frac{VPD}{{VPD}_{0}})$  Where, Γ is the leaf CO_2_ compensation point; mL is a empirical parameter; and VPD_0_ is the species-specific sensitivity to VPD. | $\propto\frac{1}{VPD}$ | $\propto VPD$ | =1.73/VPD +3.75  (R^2^=0.066, p=0.002) | =43.63VPD+24.83  (R^2^=0.20, p<0.001) |
| Exponential decay function  (Linderson et al., 2012) | WUE=$a_{0}+a_{1}e^{-a_{2}VPD}$  Where, a_0_, a_1_ and a_2_ are fitted coefficients. | $\propto e^{VPD}$ | =aVPD  +bVPD e^cVPD^ | =3.92+6.416e^-1.325VPD^  (R^2^=0.074, p=0.002) | =55.81VPD+199.81VPDe^-2.87VPD^  (R^2^=0.22, p<0.001) |
| Tree level: | | | | | |
| Reciprocal function  (Lindroth and Cienciala, 1996) | WUE = k/VPD  Where, k is a fitted coefficient. | $\propto\frac{1}{VPD}$ | n/a | =1.43/VPD -0.77  (R^2^=0.41, p=0.04) | n/a |
| Ecosystem/canopy level: | | | | | |
| Exponential decay function  (Linderson et al., 2012; Tang et al., 2006) | WUE=$a_{0}+a_{1}e^{-a_{2}VPD}$  Where, a_0_, a_1_ and a_2_ are fitted coefficients. | $\propto e^{-VPD}$ | n/a | =-3.78+6.83e^-0.49VPD^  (R^2^=0.63, p=0.0003) | n/a |
| Reciprocal function &  RESCAP model  (Baldocchi et al., 1987; Dewar, 1997; Lindroth and Cienciala, 1996) | WUE = K/VPD  Where, K is a constant that depends on leaf and atmospheric CO_2_ concentrations, sunlit and fully transpiring leaf area and partial pressure of air CO_2_. | $\propto\frac{1}{VPD}$ | n/a | =0.38/VPD +0.50  (R^2^=0.43, p=0.0003) | n/a |

Table S1 Summary of formulations and goodness of fit of the responses of WUE and WUEi at the multiple spatial scales to VPD

(Notes: R^2^ is the ratio of the explained sum of squares to the total sum of squares; a, b, c are fitted constants.)

Table S2. Three-way ANOVA test on leaf-level WUE and iWUE

| **Sources** | | **numDF** | **Sum Square** | **Mean Square** | **F-value** | **p-value** |
| --- | --- | --- | --- | --- | --- | --- |
| **Leaf-level WUE** | |  |  |  |  |  |
|  | *Model formula: aov( leaf-level WUE~ Thinning*Aspect*Date)* | | | | | |
| Thinning | | 1 | 4 | 3.9 | 0.534 | 0.465 |
| Aspect | | 1 | 2 | 2.3 | 0.317 | 0.573 |
| Date | | 1 | 1413 | 1412.9 | 193.612 | <0.001 |
| Thinning × Aspect | | 1 | 0 | 0.4 | 0.059 | 0.808 |
| Thinning × Date | | 1 | 0 | 0.4 | 0.048 | 0.826 |
| Aspect × Date | | 1 | 8 | 8.5 | 1.160 | 0.282 |
| Thinning × Aspect × Date | | 1 | 1 | 0.6 | 0.077 | 0.781 |
| Residuals | | 538 | 3926 | 7.3 |  |  |
|  | |  |  |  |  |  |
| **Leaf-level WUEi** | |  |  |  |  |  |
|  | *Model formula: aov( leaf-level WUEi~ Thinning*Aspect*Date)* | | | | | |
| Thinning | | 1 | 89634 | 89634 | 43.709 | <0.001 |
| Aspect | | 1 | 10 | 10 | 0.005 | 0.946 |
| Date | | 1 | 271598 | 271598 | 132.443 | <0.001 |
| Thinning × Aspect | | 1 | 468 | 468 | 0.228 | 0.633 |
| Thinning × Date | | 1 | 4540 | 4540 | 2.214 | 0.137 |
| Aspect × Date | | 1 | 1221 | 1221 | 0.595 | 0.441 |
| Thinning × Aspect × Date | | 1 | 5 | 5 | 0.003 | 0.959 |
| Residuals | | 538 | 1103266 | 2051 |  |  |

Table S3. Three-way ANOVA test on leaf-level WUE and iWUE

| **Sources** | | **numDF** | **Sum Square** | **Mean Square** | **F-value** | **p-value** |
| --- | --- | --- | --- | --- | --- | --- |
| **Leaf-level WUE** | |  |  |  |  |  |
|  | *Model formula: aov( leaf-level WUE~ Thinning *Date)* | | | | | |
| Thinning | | 2 | 9.4 | 4.7 | 0.436 | 0.648 |
| Date | | 1 | 335.4 | 335.4 | 31.195 | <0.001 |
| Thinning × Date | | 2 | 38.4 | 19.2 | 1.788 | 0.171 |
| Residuals | | 142 | 1526.9 | 10.8 |  |  |
|  | |  |  |  |  |  |
| **Leaf-level WUEi** | |  |  |  |  |  |
|  | *Model formula: aov( leaf-level WUEi~ Thinning*Date)* | | | | | |
| Thinning | | 2 | 42440 | 21220 | 11.18 | <0.001 |
| Date | | 1 | 66704 | 66704 | 35.15 | <0.001 |
| Thinning × Date | | 2 | 3986 | 1993 | 1.05 | 0.353 |
| Residuals | | 142 | 269449 | 1898 |  |  |
|  | |  |  |  |  |  |
| **Leaf-level photosynthesis** | |  |  |  |  |  |
|  | *Model formula: aov( leaf-level photosynthesis~ Thinning*Date)* | | | | | |
| Thinning | | 2 | 4682 | 2341.2 | 15.464 | <0.001 |
| Date | | 1 | 629 | 628.8 | 4.154 | 0.04 |
| Thinning × Date | | 2 | 13 | 6.6 | 0.044 | 0.957 |
| Residuals | | 142 | 21498 | 151.4 |  |  |
|  | |  |  |  |  |  |
| **Leaf-level transpiration** | |  |  |  |  |  |
|  | *Model formula: aov( leaf-level transpiration~ Thinning*Date)* | | | | | |
| Thinning | | 2 | 304.9 | 152.5 | 29.666 | <0.001 |
| Date | | 1 | 389.2 | 389.2 | 75.733 | <0.001 |
| Thinning × Date | | 2 | 10.7 | 5.4 | 1.046 | 0.354 |
| Residuals | | 142 | 729.7 | 5.1 |  |  |
|  | |  |  |  |  |  |
| **Leaf-level stomatal conductance** | |  |  |  |  |  |
|  | *Model formula: aov( leaf-level stomatal conductance~ Thinning*Date)* | | | | | |
| Thinning | | 2 | 369 | 184.43 | 3.163 | 0.045 |
| Date | | 1 | 298 | 298.01 | 5.110 | 0.025 |
| Thinning × Date | | 2 | 369 | 184.56 | 3.165 | 0.045 |
| Residuals | | 142 | 8281 | 58.32 |  |  |

Note: Leaf biomass for each leaf-level measurement was visually controlled by selecting tree branches with similar size and numbers of needles to minimize the errors due to variances in leaf biomass.

Table S4. ANCOVA test on tree-level WUE and mean daily tree transpiration

| Test variable: tree-level WUE | | | | | |
| --- | --- | --- | --- | --- | --- |
| Model structure: Intercept + Initial DBH + Thinning + Year + Thinning * Year | | | | | |
| Source | Type III Sum of Squares | df | Mean Square | F | Sig. |
| Corrected Model | 6.161^a^ | 6 | 1.027 | 3.049 | .028 |
| Intercept | 5.279 | 1 | 5.279 | 15.676 | .001 |
| Initial DBH | 3.525 | 1 | 3.525 | 10.467 | .004 |
| Thinning | 2.960 | 2 | 1.480 | 4.395 | .026 |
| Year | .119 | 1 | .119 | .352 | .559 |
| Thinning * Year | .119 | 2 | .060 | .177 | .839 |
| Error | 6.735 | 20 | .337 |  |  |
| Total | 18.081 | 27 |  |  |  |
| Corrected Total | 12.896 | 26 |  |  |  |
| Note:  a. R Squared = .478 (Adjusted R Squared = .321)  Levene's Test of Equality of Error Variances p =.261.  There were not significant differences between initial DBH of trees among NT, T1, and T2. (p=0.610). | | | | | |
|  | | | | | |
|  | | | | | |
| Test variable: tree BAI | | | | | |
| Model structure: Intercept + Initial DBH + Thinning + Year + Thinning * Year | | | | | |
| Source | Type III Sum of Squares | df | Mean Square | F | Sig. |
| Corrected Model | 29.082^a^ | 6 | 4.847 | 2.721 | .041 |
| Intercept | 9.479 | 1 | 9.479 | 5.321 | .031 |
| Initial DBH | 3.096 | 1 | 3.096 | 1.738 | .202 |
| Thinning | 25.071 | 2 | 12.535 | 7.037 | .005 |
| Year | .545 | 1 | .545 | .306 | .586 |
| Thinning * Year | 2.661 | 2 | 1.331 | .747 | .486 |
| Error | 37.410 | 21 | 1.781 |  |  |
| Total | 115.151 | 28 |  |  |  |
| Corrected Total | 66.492 | 27 |  |  |  |
| Note:  a. R Squared = .437 (Adjusted R Squared = .277)  Levene's Test of Equality of Error Variances p =.102. | | | | | |
|  | | | | | |
| Test variable: mean daily tree transpiration | | | | | |
| Model structure: Intercept + Initial DBH + Thinning + Year + Thinning * Year | | | | | |
| Source | Type III Sum of Squares | df | Mean Square | F | Sig. |
| Corrected Model | 75.009^a^ | 6 | 12.502 | 8.163 | .000 |
| Intercept | 8.297 | 1 | 8.297 | 5.418 | .031 |
| Initial DBH | 37.928 | 1 | 37.928 | 24.765 | .000 |
| Thinning | 19.009 | 2 | 9.505 | 6.206 | .008 |
| Year | 5.032 | 1 | 5.032 | 3.286 | .085 |
| Thinning * Year | 1.099 | 2 | .550 | .359 | .703 |
| Error | 30.630 | 20 | 1.532 |  |  |
| Total | 362.087 | 27 |  |  |  |
| Corrected Total | 105.639 | 26 |  |  |  |
| Note:  a. R Squared = .983 (Adjusted R Squared = .978)  Levene's Test of Equality of Error Variances p =0.858.  There were not significant differences between initial DBH of trees among NT, T1, and T2. (p=0.610). | | | | | |

Note: BAI record of the tree No. 10 was missing. Sap flow probe on the tree No.3 malfunctioned in the growing season of 2017.


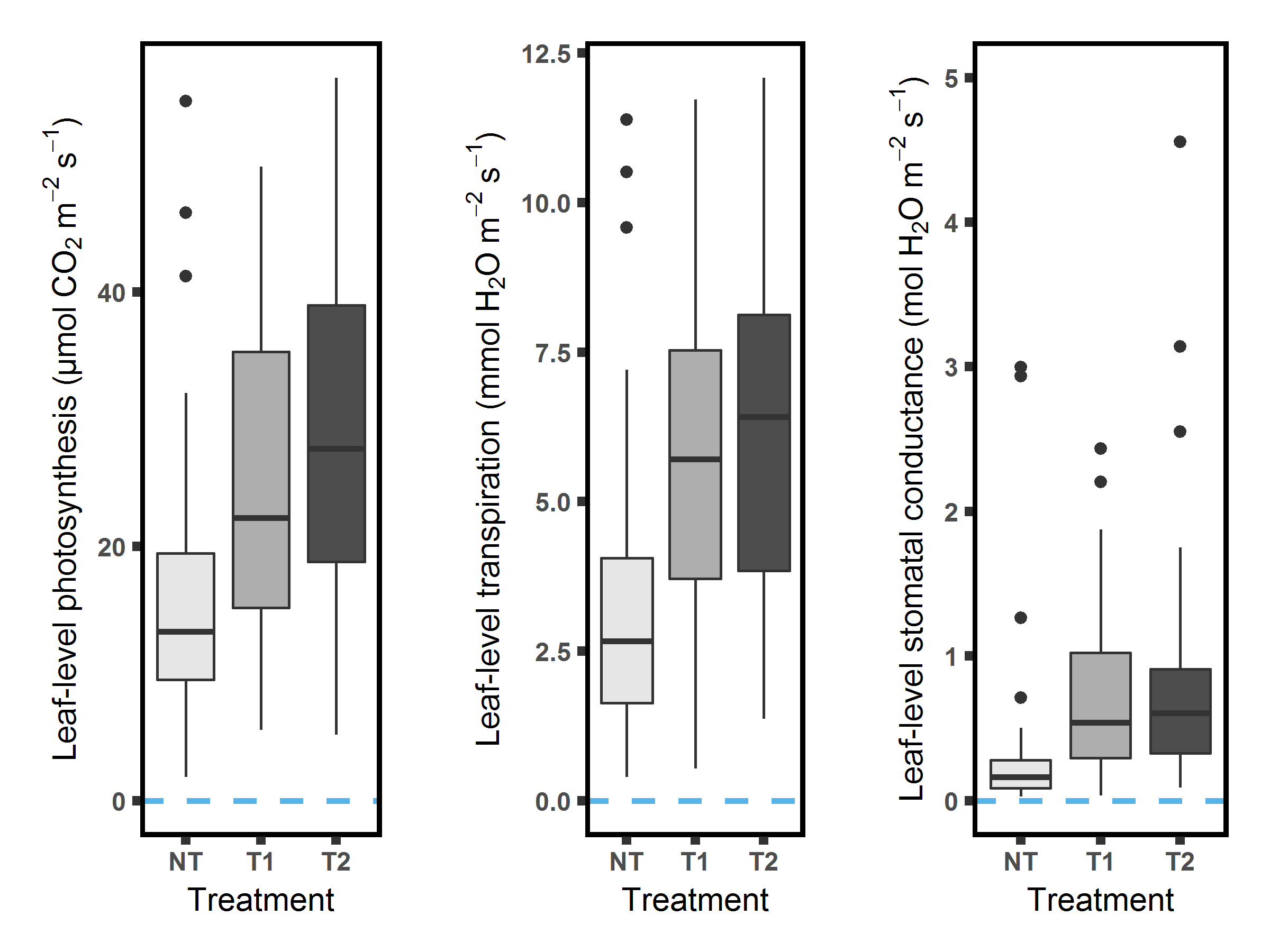


Figure S1. Leaf photosynthesis, transpiration and stomatal conductance during the growing season of 2017.


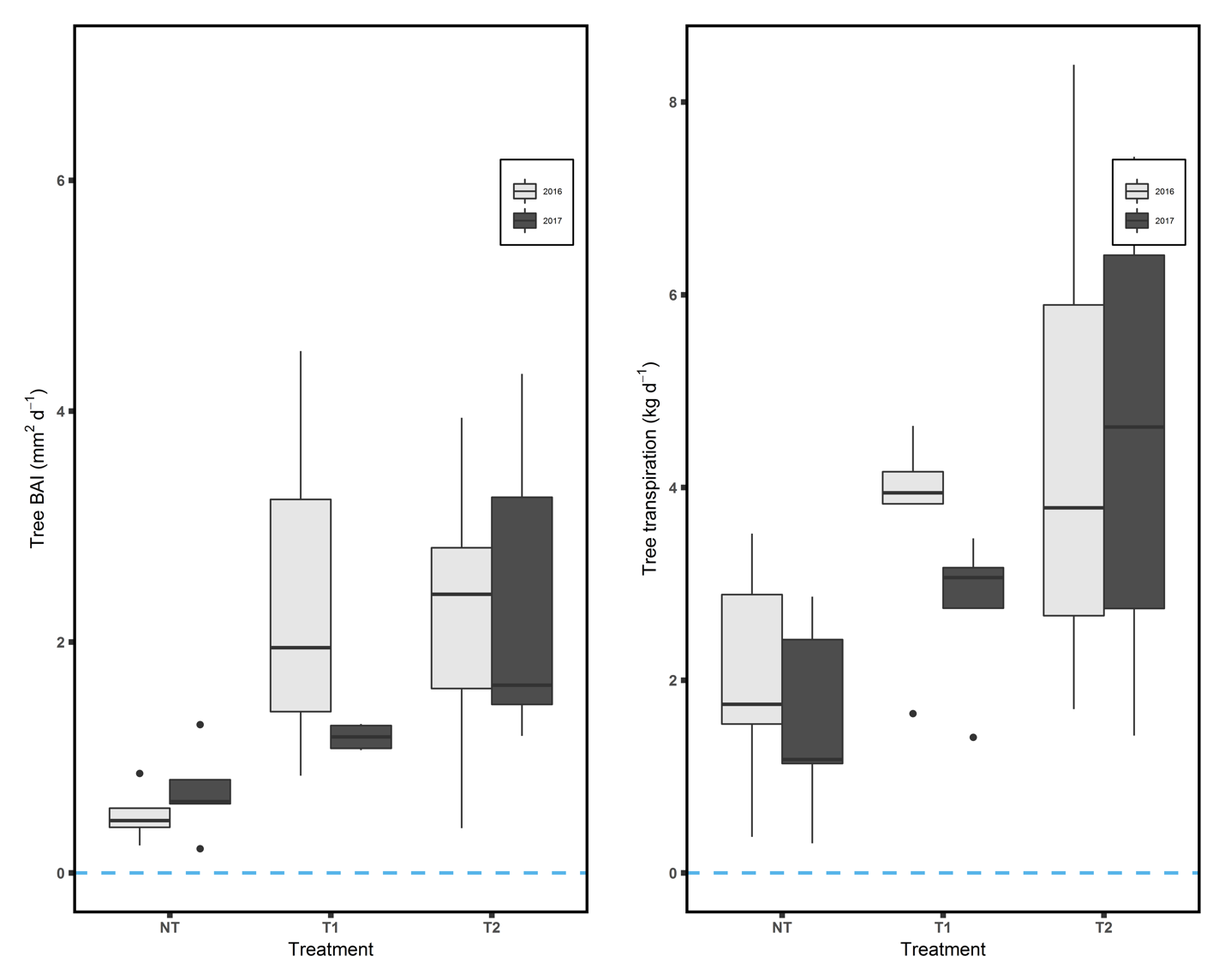


Figure S2. Mean daily basal area increment (BAI) and mean daily tree transpiration during the growing season of 2016 and 2017.


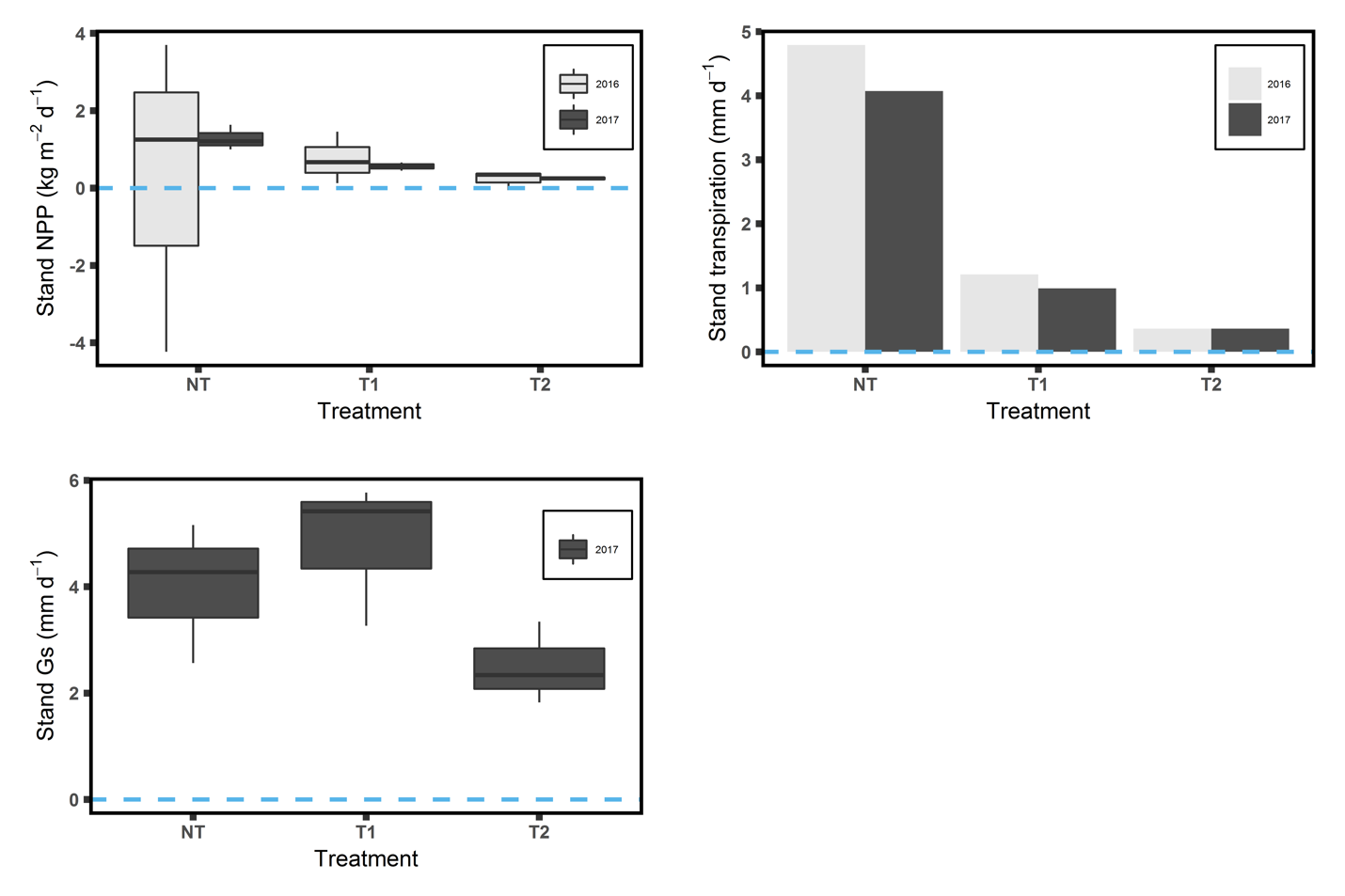


Figure. S3. Mean daily stand accumulated net primary production (ANPP) and daily stand transpiration in 2016 and 2017, and daily stand canopy conductance (Gs) in 2017.


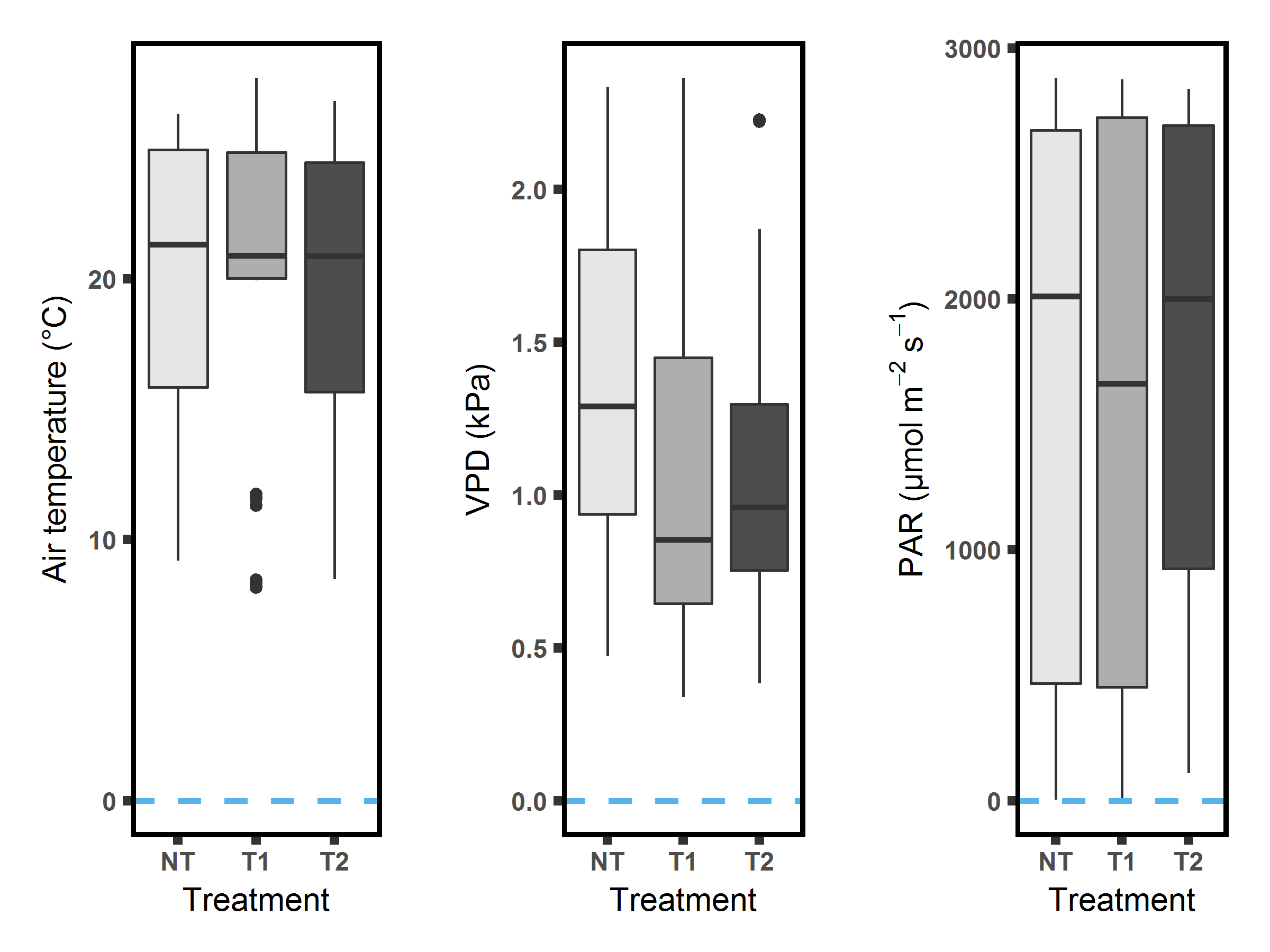


Figure S4. Temperature, VPD and PAR in NT, T1 and T2 during leaf measurement periods.


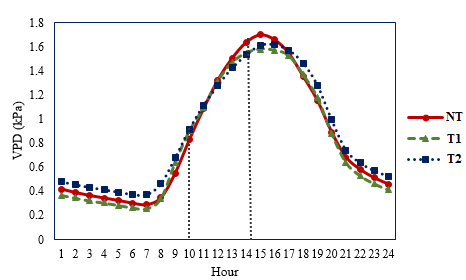


Figure S5. Daily VPD variations in NT, T1 and T2 from climate stations. Leaf-level measurements took place between 10 am to 14 pm.

References

Baldocchi, D.D., Verma, S.B. and Anderson, D.E., 1987. Canopy Photosynthesis and Water-Use Efficiency in a Deciduous Forest. Journal of Applied Ecology, 24(1): 251-260.

Dewar, R.C., 1997. A simple model of light and water use evaluated for Pinus radiata. Tree Physiology, 17(4): 259-265.

Leuning, R., 1995. A critical appraisal of a combined stomatal-photosynthesis model for C3 plants. Plant, Cell and Environment, 18(4): 339-355.

Linderson, M.-L. et al., 2012. Up-scaling of water use efficiency from leaf to canopy as based on leaf gas exchange relationships and the modeled in-canopy light distribution. Agricultural and Forest Meteorology, 152: 201–211.

Lindroth, A. and Cienciala, E., 1996. Water use efficiency of short-rotation Salix viminalis at leaf, tree and stand scales. Tree Physiology, 16(1-2): 257-262.

Lloyd, J. and Farquhar, G.D., 1994. 13C discrimination during CO2 assimilation by the terrestrial biosphere. Oecologia, 99(3): 201-215.

Tang, J. et al., 2006. Sap flux-upscaled canopy transpiration, stomatal conductance, and water use efficiency in an old growth forest in the Great Lakes region of the United States. Journal of Geophysical Research: Biogeosciences, 111(G2): n/a-n/a.

Wong, S. and Dunin, F., 1987. Photosynthesis and Transpiration of Trees in a Eucalypt Forest Stand: CO<sub>2</sub>, Light and Humidity Responses. Functional Plant Biology, 14(6): 619-632.
